# Supplementary material for: Disulfide Bond Engineering of an Endoglucanase from Penicillium verruculosum to Improve Its Thermostability
Source: Int J Mol Sci. 2019 Mar 30;20(7):1602. doi: 10.3390/ijms20071602 (PMC6479618; doi:10.3390/ijms20071602)
Supplement: Supplementary file 1 [file ijms-20-01602-s001.pdf]

## Supplementary Materials

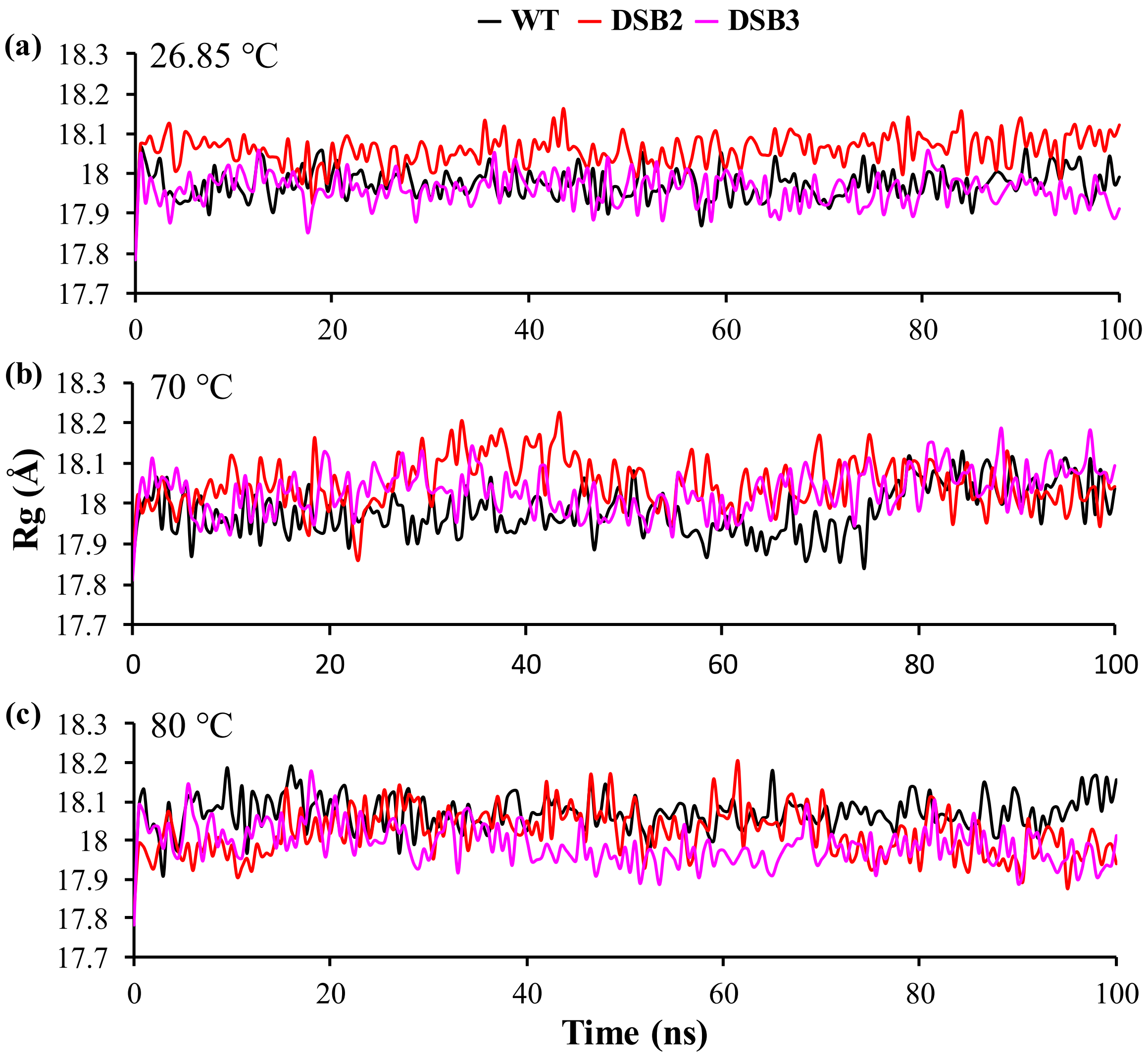


**Figure 1.** Structural stability of endoglucanase based on $R_g$. **(a)** and **(b)** At 26.85 °C and 70 °C, $R_g$ of protein indicated that variants and EGLII-wt have similar structural stability. **(c)** At 80 °C, $R_g$ of EGLII-wt showed instability whereas structures remain stable in the both variants.

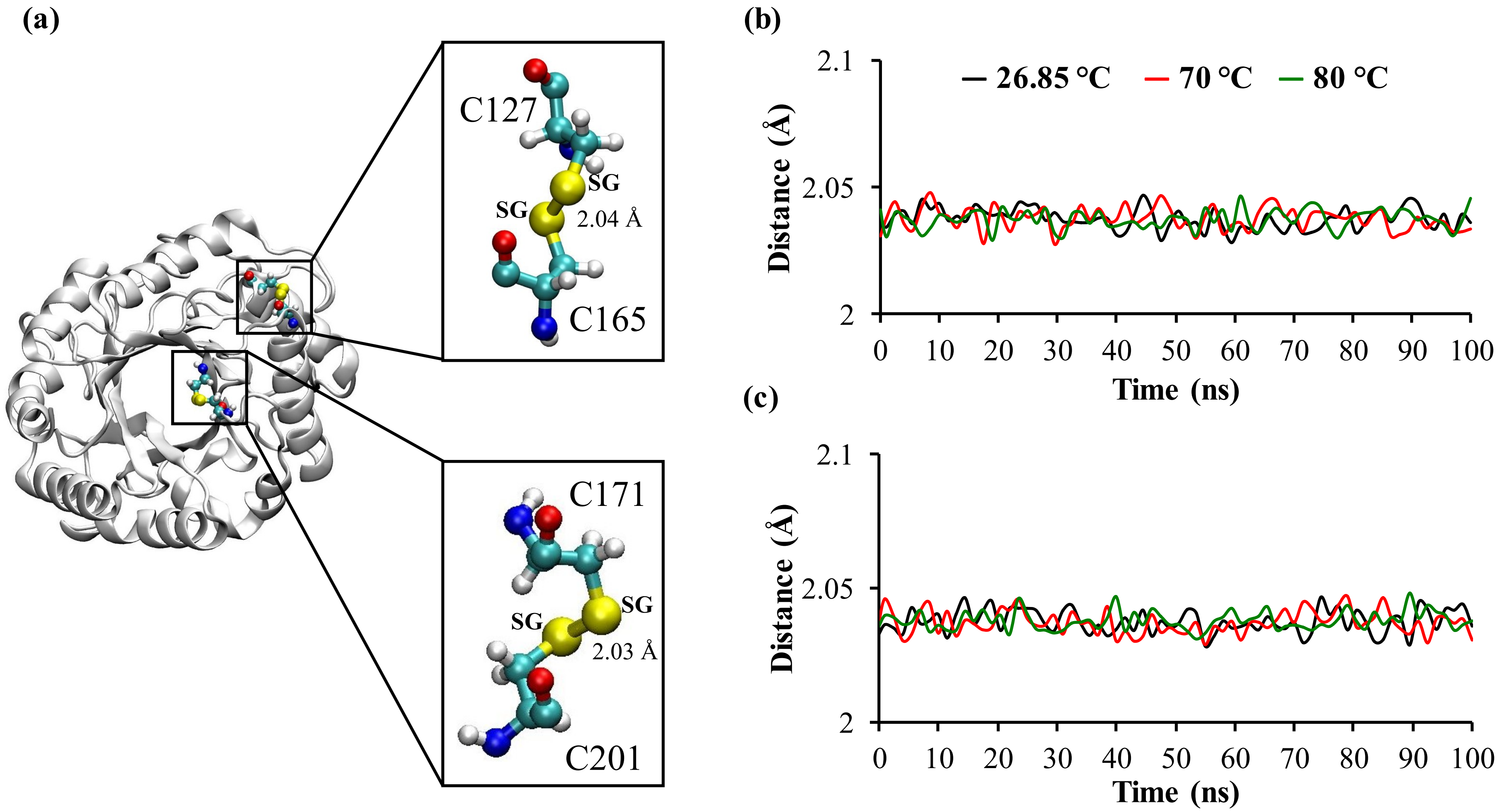


**Figure 2.** Distance stability of DSBs in variants. **(a)** The -S-S- distance (2.04 Å) in the variants DSB2 and DSB3 before simulations. (**b)** and **(c**) 100 ns simulations showed that the -S-S- distance remains stable within 2.15 Å for variants DSB2 and DSB3, respectively.
